# Supplementary material for: The Construction and Exploration of a Comprehensive MicroRNA Centered Regulatory Network in Foxtail Millet (Setaria italica L.)
Source: Front Plant Sci. 2022 May 6;13:848474. doi: 10.3389/fpls.2022.848474 (PMC9121102; doi:10.3389/fpls.2022.848474)
Supplement: Supplementary file 1 [file Data_Sheet_1.zip › Sit-miRNovel2_Seita.5G323500.1_1271_TPlot.pdf]

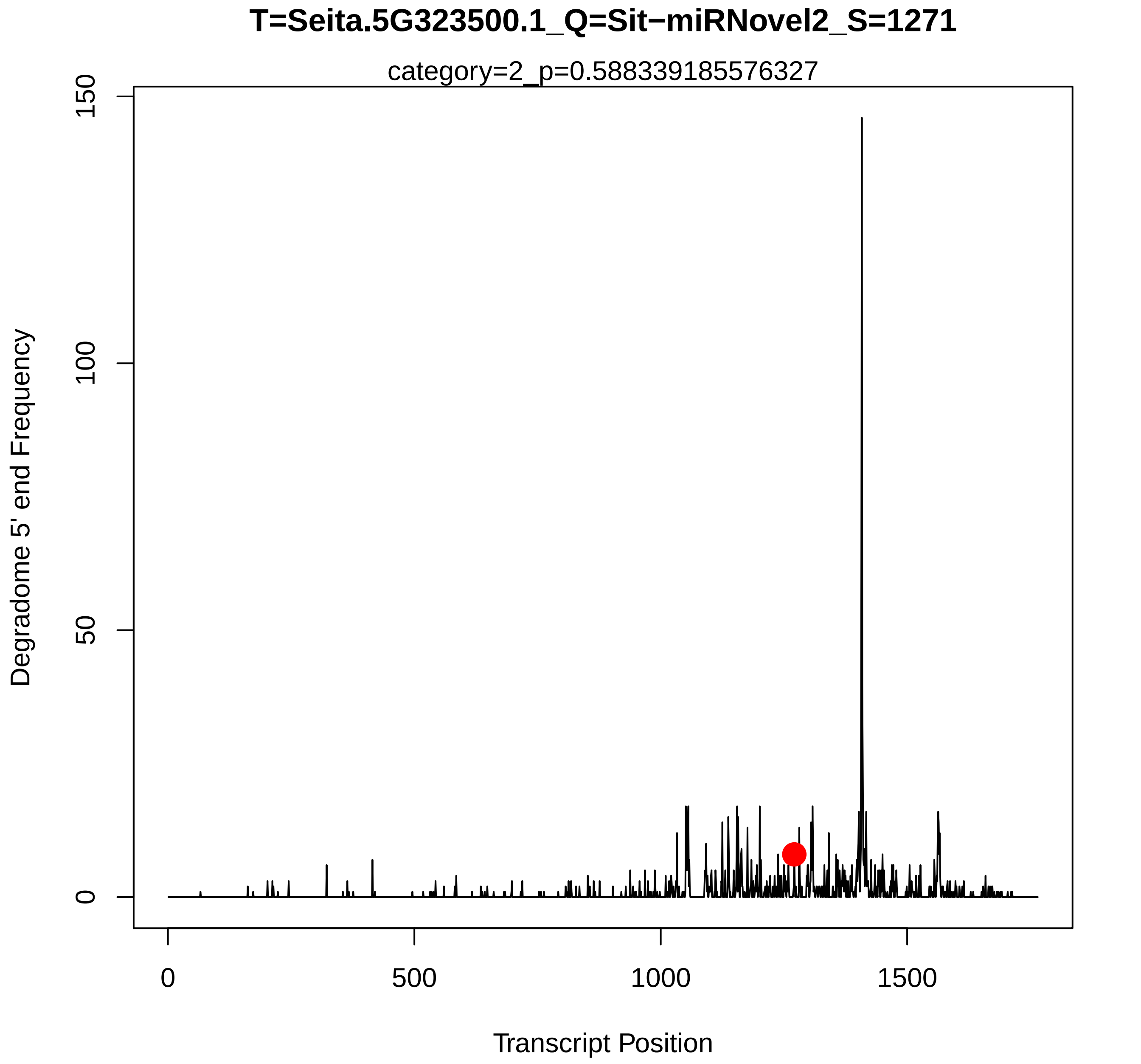

T=Seita.5G323500.1_Q=Sit−miRNovel2_S=1271
category=2_p=0.588339185576327
Degradome 5' end Frequency
Transcript Position
0
50
100
150
0
500
1000
1500
